# Quantitative Characterization of Collagen in the Fibrotic Capsule Surrounding Implanted Polymeric Microparticles through Second Harmonic Generation Imaging

**DOI:** 10.1371/journal.pone.0130386

**Published:** 2015-06-30

**Authors:** Dana Akilbekova, Kaitlin M. Bratlie

**Affiliations:** 1 Department of Materials Science & Engineering, Iowa State University, Ames, Iowa 50011, United States of America; 2 Department of Chemical & Biological Engineering, Iowa State University, Ames, Iowa 50011, United States of America; 3 Ames National Laboratory, Ames, Iowa 50011, United States of America; Pennsylvania State Hershey College of Medicine, UNITED STATES

## Abstract

The collagenous capsule formed around an implant will ultimately determine the nature of its *in vivo* fate. To provide a better understanding of how surface modifications can alter the collagen orientation and composition in the fibrotic capsule, we used second harmonic generation (SHG) microscopy to evaluate collagen organization and structure generated in mice subcutaneously injected with chemically functionalized polystyrene particles. SHG is sensitive to the orientation of a molecule, making it a powerful tool for measuring the alignment of collagen fibers. Additionally, SHG arises from the second order susceptibility of the interrogated molecule in response to the electric field. Variation in these tensor components distinguishes different molecular sources of SHG, providing collagen type specificity. Here, we demonstrated the ability of SHG to differentiate collagen type I and type III quantitatively and used this method to examine fibrous capsules of implanted polystyrene particles. Data presented in this work shows a wide range of collagen fiber orientations and collagen compositions in response to surface functionalized polystyrene particles. Dimethylamino functionalized particles were able to form a thin collagenous matrix resembling healthy skin. These findings have the potential to improve the fundamental understanding of how material properties influence collagen organization and composition quantitatively.

## Introduction

Implantation of biomaterials into the body triggers the foreign body response. Here, injured tissue attempts to stop blood loss, prevent inflammation, and restore normal function [1–4]. In the first stage, blood proteins are absorbed to the biomaterial surface, forming a provisional matrix consisting largely of fibrin [1,5,6]. Neutrophils are the first cell type to arrive to the site of the injury and characterize acute inflammation. Acute inflammation resolves quickly and is follwed by chronic inflammation. During chronic inflammation monocytes/macrophages migrate to the wound site in response to various chemokines and chemattractants such as transforming growth factor-β (TGF-β), platelet-derived growth factor, and interleukin-1 [7]. After chronic inflammation is resolved, fibroblast cells infiltrate in response to growth factors and differentiate into myofibroblasts by TGF-β activation [8,9]. Fibroblasts and myofibroblasts secrete collagen type I and III to form a fibrous capsule around the implant and contract the wound edges. A well-oriented collagen capsule around the implant has low vascular density and can hinder the function of encapsulated cells for tissue engineering applications or for artificial organs [10].

In healthy skin tissue, collagen type I is more prevalent compared to type III. In adult skin, collagen type III is about 30% of the total collagen content [11]. During the wound healing process collagen type III content can increase up to 90% [12]. Collagen type III plays an important role in regulating collagen fibrillogenesis and fibril size along with the physical properties of the tissues [13,14]. It was found that a decrease in collagen type III can lead to excessive scar formation [15]. The influence of material chemistry on collagen type III during the remodeling process is not clear and there is a need for a better understanding of the wound healing mechanisms and how these materials influence the deposition of collagen types I and III [7,15,16]. During the remodeling phase of the wound healing process collagen fibers are organized in a manner to mimic normal tissues [17]. However, healed tissue can only achieve 80% of the original strength [7]. In the resulting scar tissue collagen fibers are organized into bundles of collagen that are aligned parallel to one another while in healthy tissue collagen fibers have an isotropic orientation [18]. During fibrous capsule formation collagen type III is initially secreted by fibroblast cells and is gradually replaced by collagen type I, which becomes the main collagen type in the fibrous capsule [19]. It was shown that collagen type III is present in the fibrous capsule around the implant for 3–6 months. After 18 months for biocompatible materials there is almost no trace of collagen type III [20]. Achieving a thin capsule with randomly oriented collagen fibers and optimizing the ratio of collagen type III to collagen type I with time in the capsular matrix would be an improvement in minimizing the foreign body response to implanted biomaterials [20,21].

SHG microscopy has gained traction in biomedical imaging for measuring ordered biomolecular assemblies [22,23]. The nonlinear interaction of incident laser beams with non-centrosymmetric structures leads to the generation of new photons with twice the energy or exactly half of the wavelength of the incident light [24]. The SHG process involves molecules being excited by two photons of light, having the same frequency, to a virtual state, which results in the absence of photobleaching and photodamage [25]. Some of the main applications of SHG microscopy have been imaging collagen fibers, determining fiber organization, and parameters related to the second-order nonlinear susceptibility due to the triple helical structure of collagen fibers. The susceptibility tensor elements are related to the material properties and form the basis for contrasting different second harmonic generating molecules [25–28]. Chen *et al*. showed that variation of the susceptibility tensor elements could be used for differentiating between molecular sources of SHG. A polarization resolved SHG microscope was used for examining the dermis of normal human skin to generate a spatial map of the susceptibility tensor components throughout the tissue sample. The ratio of the tensor elements exhibited two distinct peaks corresponding to collagen types I and III. The ratio of these peaks accurately estimated the collagen type I and III concentration in the tissues [29]. Another advantage of SHG imaging of collagen is that it is label free and does not require additional sample preparation. In comparison, sample preparation for histology can take hours to days.

Here, SHG imaging was used for quantitative characterization of collagen fibers deposited in response to implanted surface modified polystyrene microspheres. In this paper we investigated how surface modified polystyrene beads influence the final outcome of the foreign body response, namely fibrosis, through quantification of collagen fiber orientation and structural inhomogeneities in the fibrotic capsule surrounding the particles.

## Materials and Methods

### Materials

Polystyrene beads (0.93 μm diameter, Magsphere, Inc., Pasadena, CA), poly(methyl methacrylate) coated polystyrene beads (PMMA, 0.79 µm diameter, Magsphere, Inc.), carboxylated polystyrene beads (0.75 µm diameter, Magsphere, Inc.), VBC (vinylbenzylchlroide) polystyrene beads (1.0 μm diameter, Magsphere, Inc.), aldehyde/sulfate polystyrene beads (0.96 μm diameter, Life Technologies, Grand Island, NY), amidine polystyrene (1.0 μm diameter, Life Technologies), amino-polystyrene particles (0.91 μm diameter, Spherotech, Inc., Lake Forest, IL), hydroxyl polystyrene particles (0.79 μm diameter, Spherotech, Inc.), sulfonate polystyrene particles (0.92 μm diameter, Spherotech, Inc.), dimethylamino polystyrene particles (0.85 μm diameter, Spherotech, Inc.).

### Ethics statement

The research protocol was approved by the local animal ethics committee at Iowa State University (Institutional Animal Care and Use Committee) prior to initiation of the study.

### Animals

Six week old female SKH1-E mice were obtained from Charles River Laboratories (Wilmington, MA). The mice were maintained at the animal facilities of Iowa State University, accredited by the American Association of Laboratory Animal Care, and were housed under standard conditions with a 12-hour light/dark cycle. Both water and food were provided *ad libitum*.

### Injections

Injections were performed in accordance with ISO 10993–6:2007. Prior to injection all materials were sterilized. Polystyrene particles were sterilized by washing three times in 70% ethanol, followed by three washes in deionized sterile water by centrifuging at 10,000 g for 3 minutes. The particles were re-suspended to a concentration of 2%-w/v. The mice were anesthetized via isoflurane inhalation at a concentration of 1–4% isoflurane/balance O_2_ to minimize movement. Their backs were scrubbed with 70% isopropyl alcohol and the animals were subcutaneously injected with 100 µL of 2%-w/v polystyrene beads on the mouse’s back. All experiments were conducted in quintuplicate. Control samples of tissue that were not injected with particles were also obtained.

### Histology

Mice were euthanized via CO_2_ asphyxiation 28 days after subcutaneous injection. The injected biomaterials and surrounding tissue were excised. The tissues were then fixed in 10% formalin, embedded in paraffin, cut into 5 µm sections, and stained using Masson’s Trichrome stain for histological analysis. Fibrotic capsule thickness was measured through ImageJ (NIH, Bethesda, MD). The data presented is the mean of the five injected replicates, each of which was measured a minimum of four times.

### Immunohistochemistry

Tissue sections were deparaffinized and were heated to 120˚C at 15 psi for 10 minutes in sodium citrate buffer (10 mM sodium citrate, 0.05% Tween 20, pH 6.0). Tissue sections were stained with 1:100 goat polyclonal antibody against collagen type I (sc-25974, Santa Cruz Biotechnology, Dallas, TX) and 1:100 rabbit polyclonal to collagen type III (Abcam, Cambridge, MA) overnight. Secondary antibodies of 1:200 donkey anti-goat IgG fluorescein isothiocyanate (sc-2024, Santa Cruz Biotechnology) and 1:600 anti-rabbit antibody Alexa Fluor 594 (ab150080, Abcam, Cambridge, MA) were used for 1 hour in the dark. Then tissues were washed 3 times with phosphate buffered saline (PBS) for 5 minutes and were incubated with 0.1 μg/mL DAPI (4',6-diamidino-2-phenylindole, Sigma, St. Louis, MO) for 5 minutes. Then slides were mounted with glycerin jelly and imaged with an EVOS FLoid Imaging Station (Life Technologies, Grand Island, NY) using the blue (excitation/emission 390/446 nm), green (482/532 nm), and red (586/646 nm) channels.

### Collagen gel preparation

Collagen type I (BD Biosciences, Franklin Lakes, NJ) and collagen type III (Millipore, Billerica, MA) were solubilized in 20 mM acetic acid at 10 mg/mL. Collagen types I and III were mixed on ice with 10x PBS, 1 M NaOH, and deionized H_2_O. Solutions with 10%, 30%, 70%, and 90% of collagen type III were gelled at 37˚C for 1 h. All gels were 4 mg/mL collagen.

### Microscopy equipment

The laser system is a mode-locked Ti:Sapphire laser (100 fs pulse width, 1 kHz repetition rate, Libra, Coherent, Santa Clara, CA) that produces an 800 nm fundamental. The average power at the samples was controlled using a combination of a half-wave plate and a Glan-Thompson polarizer (Thorlabs, Newton, NJ) and the power was kept between 1–10 mW to avoid tissue damage. SHG signal was collected in the transmission mode. For this setup, an inverted microscope stand (AmScope, Irvine, CA) and 20x Nikon Plan Fluorite objective (0.50 NA, 2.1 mm, Nikon, Melville, NY) were used to focus the beam and the SHG light was collected with a 40x Nikon water immersion objective (0.8 NA, 3.5 mm, Nikon, Melville, NY). The transmitted SHG signal was reflected by a dichroic mirror (Thorlabs, Newton, NJ) and separated from the fundamental beam with a short pass filter < 450nm (Thorlabs, Newton, NJ) and 808 nm notch filter (NF-808.0-E-25.0M, Melles Griot, Rochester, NY), before detection by an intensified CCD camera (iCCD, iStar 334T, Andor, Belfast, UK). For polarization resolved SHG experiments a Glan-Thompson polarizer and a half-wave plate mounted on a motor driven rotational stage (Thorlabs Newton, NJ) were used to achieve linear polarization. Images of collagen gels and tissue sections were collected every 10° from 0° to 350°. A minimum of four images for each experimental condition was taken. Birefringence was calculated to be within the resolution of the polarization angles for the thickness of samples (5 μm) used in this study.

### Theoretical background and image processing

The SHG intensity of collagen as a function of polarization angle of the incident laser beam can be written as:

$$I_{SHG}=c\cdot \left\{{\left[{\text{sin}}^{2}\;\left(\theta_{e}-\theta_{o}\right)+\left(\frac{\chi_{zzz}}{\chi_{zxx}}\right)\;{\text{cos}}^{2}\;\left(\theta_{e}-\theta_{o}\right)\right]}^{2}+{\left(\frac{\chi_{xzx}}{\chi_{zxx}}\right)}^{2}{\text{sin}}^{2}\;\left(2\left(\theta_{e}-\theta_{o}\right)\right)\right\}$$ (1)

where $\frac{\chi_{zzz}}{\chi_{zxx}}$ and $\frac{\chi_{xzx}}{\chi_{zxx}}$ are second-order susceptibility tensor element ratios; *θ*_*e*_ and *θ*_*o*_ are the incident polarization angle and collagen fiber angle, respectively; and c is a normalization constant. Tensor elements are used as a contrast mechanism for identifying sources of SHG signal [29].

SHG images of the samples were obtained using the software package provided with the iCCD camera (Solis, Andor, Belfast, UK) with 512 × 512 active pixels. Final images were acquired by averaging at least 15 images for each polarization angle. A small background, likely due to the ambient light noise, was subtracted from all images (ImageJ, NIH, Bethesda, MD). After background subtraction, images were filtered using a median noise filter (3 × 3) to attenuate the salt and pepper noise in SHG images (ImageJ, NIH, Bethesda, MD). Matlab (MathWorks, Natick, MA) was used to determine the deposited collagen orientation angle and susceptibility tensors for every region of interest (ROI) by fitting Eq 1 with the Levenberg-Marquardt algorithm [30]. Images were binned to obtain 2 × 2 pixel areas (i.e. 2 × 2 ROIs) and Matlab was used to determine the mean signal intensity per pixel and per ROI. Photon counts below 5 counts per pixel were excluded from analysis, which was found to be below the limit of detection for this setup. The limit of detection was determined by blocking the laser light from entering the CCD camera and measuring the ambient noise detected by the CCD camera. The Matlab script also provides a histogram of the susceptibility tensor element ratios and the angle distributions.

### Collagen fiber orientation analysis

SHG radar graphs where SHG intensity was plotted as a function of incident polarization angle can be used for quantifying collagen fiber organization in response to laser irradiation at different polarization angles. Polarization anisotropy was used as one of the parameters for determining the degree of organization of the collagen fibers and was calculated using [31]:

$$PA=\frac{I_{∥}-I_{⊥}}{I_{∥}+I_{⊥}}$$ (2)

where *I*_∥_ and *I*_⊥_ are SHG intensities at horizontal and vertical polarizations, respectively. For PA = 1 or -1 collagen orientation is uniaxial, while for collagen fibers with PA = 0 the orientation is completely random.

The SHG radar plots were also Fourier transformed. This transformation was used to extract shape characteristics of SHG radar graphs. Fourier space representation of *f*(*m*, *n*) can be expressed as follows:

$$F\left(w_{1},w_{2}\right)=\sum_{m=-\infty }^{\infty }\sum_{n=-\infty }^{\infty }f\left(m,n\right)e^{jw_{1}m}e^{jw_{2}n}$$ (3)

where *w*_1_, *w*_2_ and *n*, *m* are period and space parameters. *F*(*w*_1_, *w*_2_) is the Fourier space representation of *f*(*m*, *n*). The fast Fourier transformation (FFT) algorithm was used for extracting the shape signature. Similarity measurements between the transformed data and any shape can be calculated through the following:

$$D=\sqrt{\sum_{u=1}^{M}{\left(A_{u}^{i}-A_{u}^{j}\right)}^{2}}\;\left(u=1,2,\ldots ,N-1\right)$$ (4)

Where $A_{u}^{i}$ and $A_{u}^{j}$ are normalized amplitude of the shape *i* and *j*, i.e. $A_{u}=\frac{\left\|F_{u}\right\|}{\left\|F_{0}\right\|}$, where ‖*F*_*u*_‖ is the amplitude of F(u) and ‖F_0_‖ is the amplitude of the 0^th^ Fourier descriptor. M is the number of harmonics needed to index the shape. In this case, the shape that the SHG radar plots are being compared to is a perfect circle. *D* is defined as one of the shape factors describing SHG polarimetry profiles [32]. For *D* = 1 the collagen orientation is uniaxial, while for collagen fibers with *D* = 0 the orientation is completely random.

Eccentricity is another parameter that can be used to describe a shape. In this case, the shape factor measures how much a shape deviates from being circular. The following expression was used for calculating eccentricity (*e*) of the polarimetry profiles:

$$e=\frac{a}{d}$$ (5)

Where *a* is the semimajor axis and *d* is the distance between the center and the directrix. *e* = 0 indicates perfectly circular object with collagen fibers oriented completely randomly while *e* = 1 represents a line.

### Statistical analysis

The statistical significance of the mean comparisons was determined by ANOVA using the JMP Pro statistical software package. Pair-wise comparisons were analyzed with Tukey HSD. Differences were considered statistically significant for p < 0.05.

## Results

### Linearity of SHG collagen type III tensor ratio to concentration analysis

**Fig 1:**
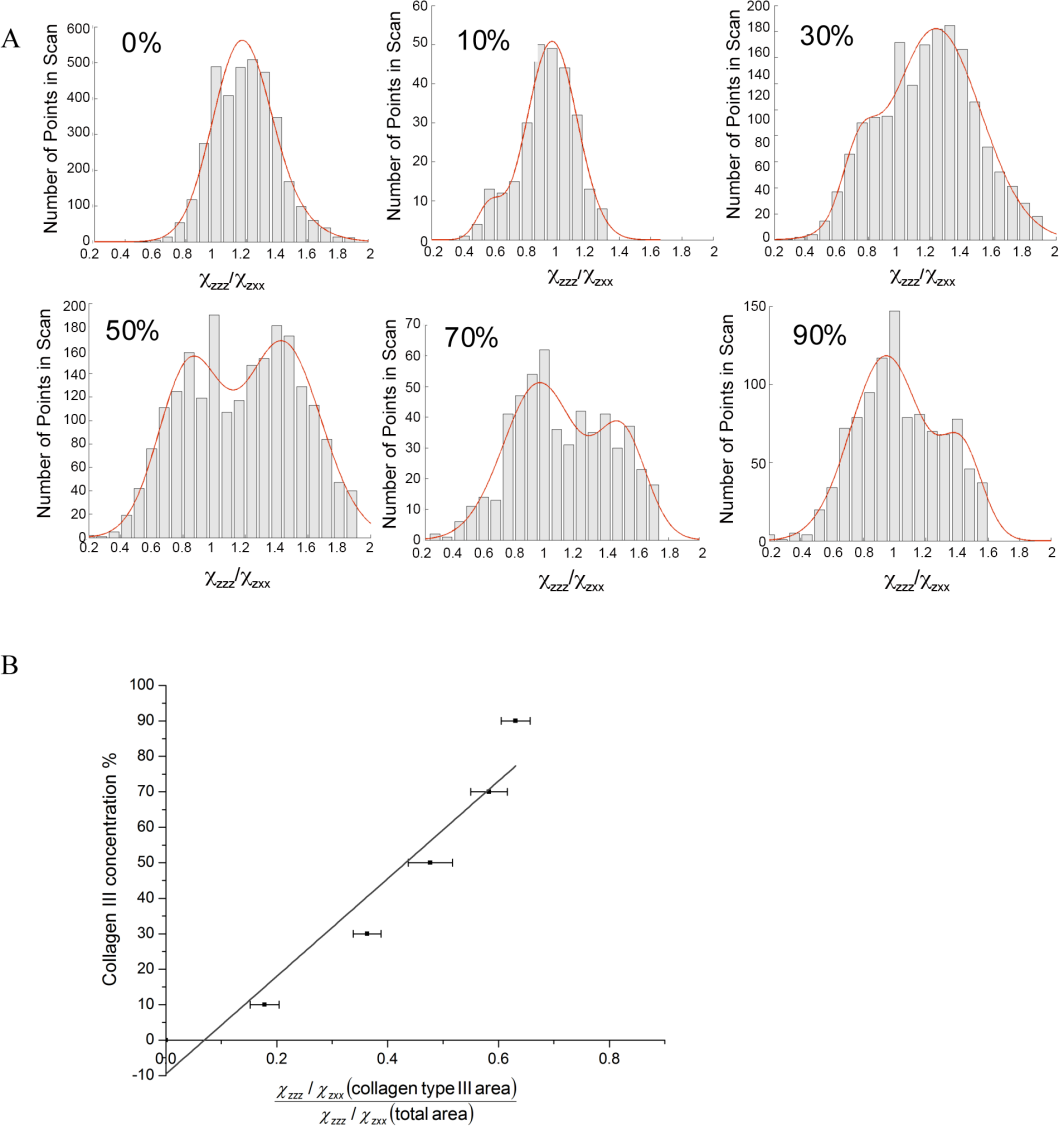
A) Histograms of $\frac{\chi_{\text{zzz}}}{\chi_{\text{zxx}}}$ values obtained for gels with varying collagen type III concentration. The bars represent experimentally acquired data. The red line is the bimodal Gaussian fit to the data. B) Standard curve for collagen type III concentration. The known concentration of collagen concentration is plotted against the ratio of the area under the collagen type III peak ~0.8 to the total area under the Gaussian fit.

Developing a standard curve to quantify the presence of collagen type III was achieved through analysis of collagen gels of varying compositions. The second order susceptibility ratio $\frac{\chi_{zzz}}{\chi_{zxx}}$ was determined for all gel mixtures and values were plotted as a histogram. The histograms and their fits are shown in Fig 1A. Since various SHG sources will results in different values of the second order susceptibility tensor elements, histogram distributions provide information about the % concentration of collagen III in gel blends. This can be further visualized by observing the appearance and increase of a second peak in the histogram as collagen type III content is increased. Based on this observation, the ratio ~0.8 is assigned to collagen type III and the ratio ~1.2 is assigned to collagen type I. These results are inline with previous assignments [32,33]. The exact histogram peak positions determined through fitting with a Gaussian are shown in S1 Table. The ratio of the area under the collagen type I assigned peak compared to the total area in the histogram was calculated for each collagen gel blend and exhibited a linear dependence (Fig 1B, R^2^ = 0.931) on collagen type III concentration, as expected.

### Collagen response to subcutaneously implanted microspheres

**Fig 2:**
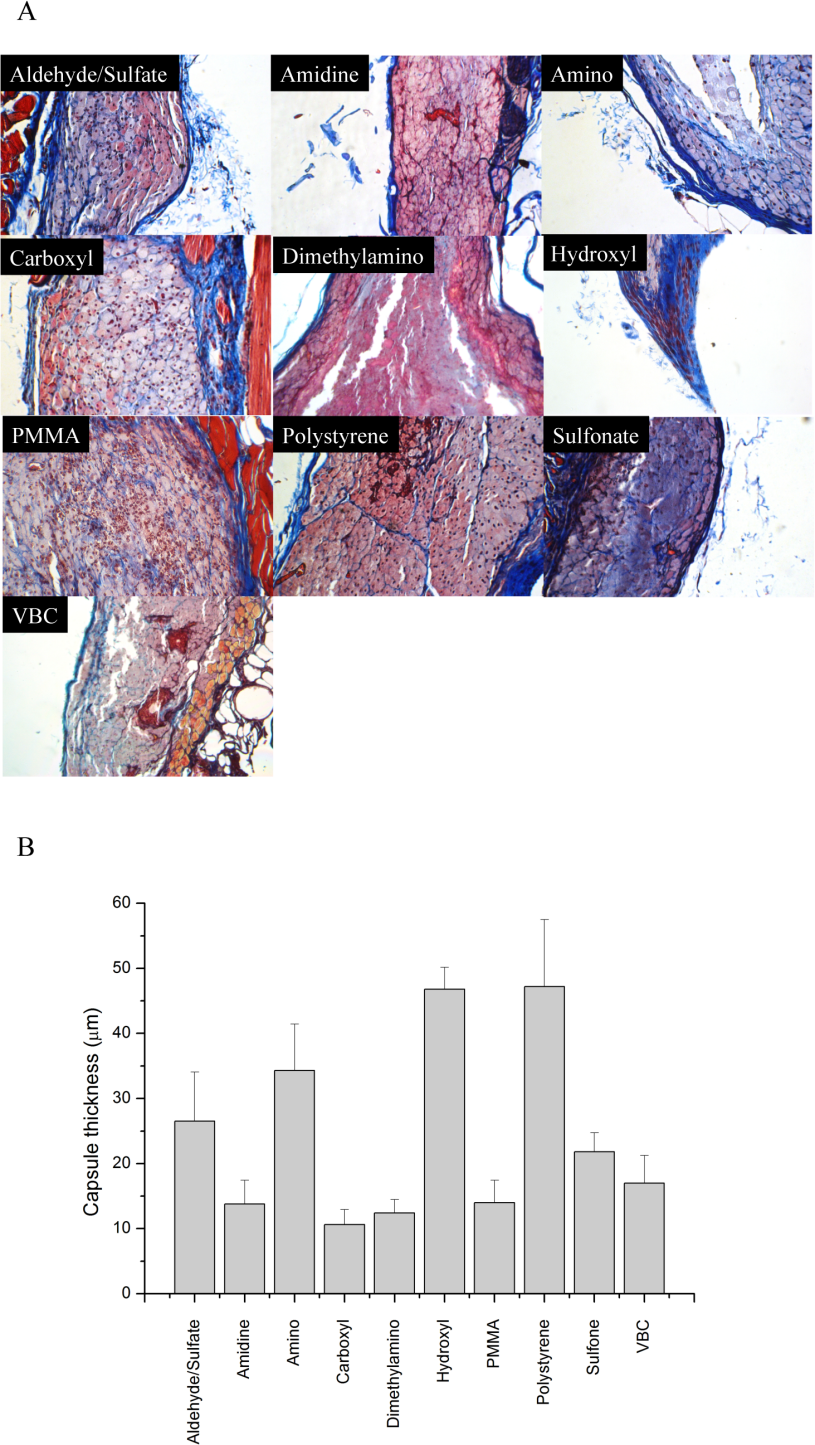
Masson’s Trichrome staining of representative sections subcutaneously injected. (A) Representative sections stained with Masson’s Trichrome are shown for the various polystyrene particles injected into mice. (Magnification 20×, images are 540 × 360 μm^2^) (B) Quantification of capsule thickness. Error bars represent the standard deviation.

Mice (SKH1-E strain) were subcutaneously injected with polystyrene particles. These particles exhibit high cellular adhesive properties, which provokes a foreign body response, ultimately resulting in a fibrotic capsule surrounding the implant [34,35]. After 28 days, the tissues were excised and stained with a Masson’s Trichrome stain in which black represents nuclei, pink denotes cytoplasm, and blue indicates collagen. Representative images are shown in Fig 2A. The injected polystyrene particles are easily phagocytized by macrophages due to their size (0.75–1.0 μm), as observed in all of the representative images. Surrounding the macrophages, a fibrotic capsule can be observed (blue). Quantification of the capsule thickness is shown in Fig 2B. The variable thickness of the fibrotic capsule surrounding implants leads to large errors (often >25% [36,37]) in quantification.

**Fig 3:**
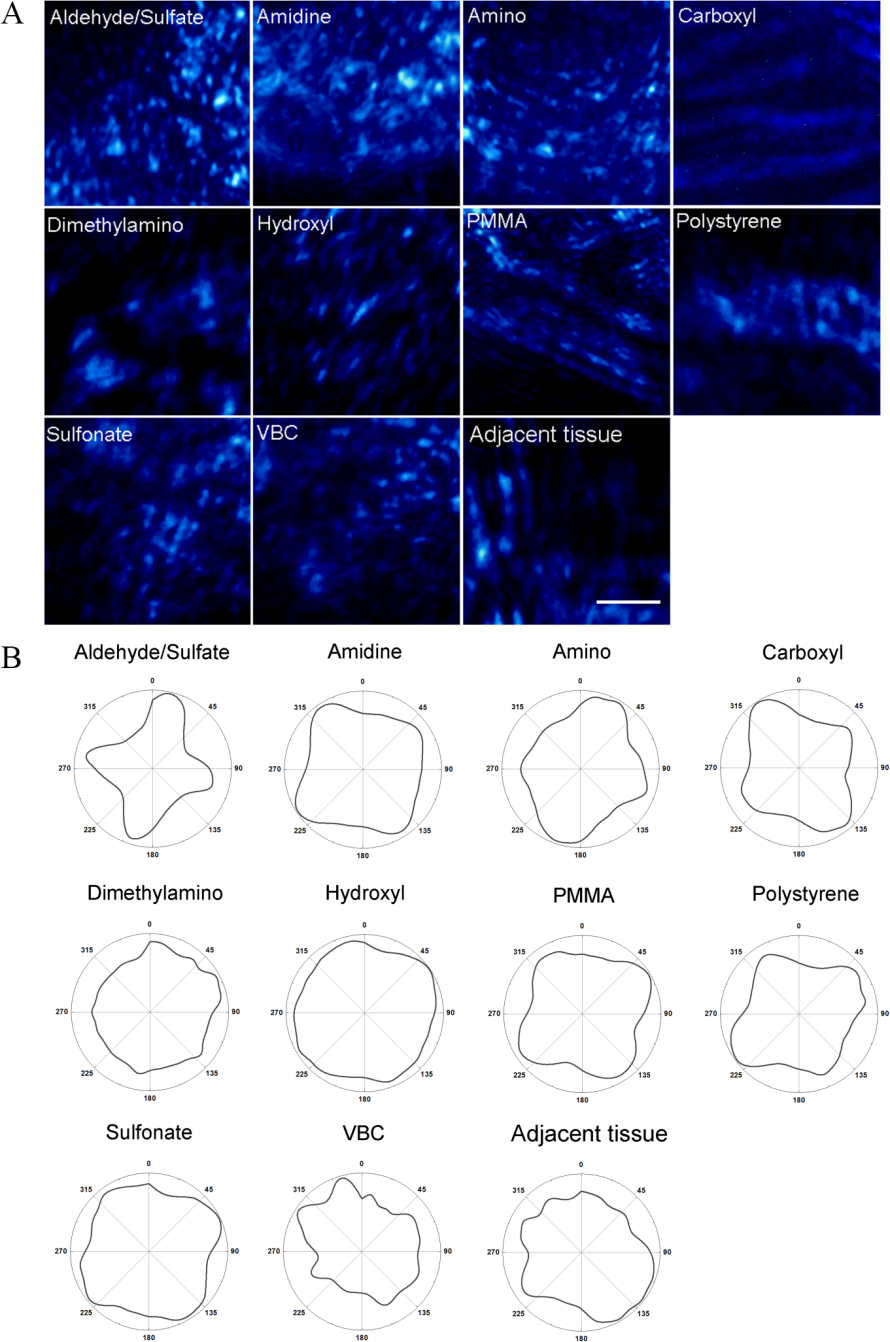
A) Representative SHG images of the fibrotic capsule in tissue section with different subcutaneously implanted polystyrene particles. SHG intensity is in blue pseudocolor. The scale bar represents 20μm. B) SHG polarimetry profiles for tissue sections with on different latex beads. The y-axis corresponds to SHG intensity in arbitrary units and the azimuthal angles correspond to polarization angles.

Collagen fiber orientation and composition in the fibrotic capsule surrounding the particles were quantified through SHG. Representative images obtained from SHG are shown in Fig 3A. Polarimetry profiles of SHG as a radar graph show the collagen distribution in tissue sections (Fig 3B). These profiles were generated by integrating the image intensity at each polarization angle. In examining the shapes of the polarimetry profiles, it appears that tissue sections with aldehyde/sulfate, amidine, amino, carboxyl, and polystyrene particles have a higher degree of collagen organization compared to the other particles and the control section. Dimethylamino and sulfonate particles exhibited a round shaped polarimetry profiles indicating a completely random distribution of collagen fibers.

**Fig 4:**
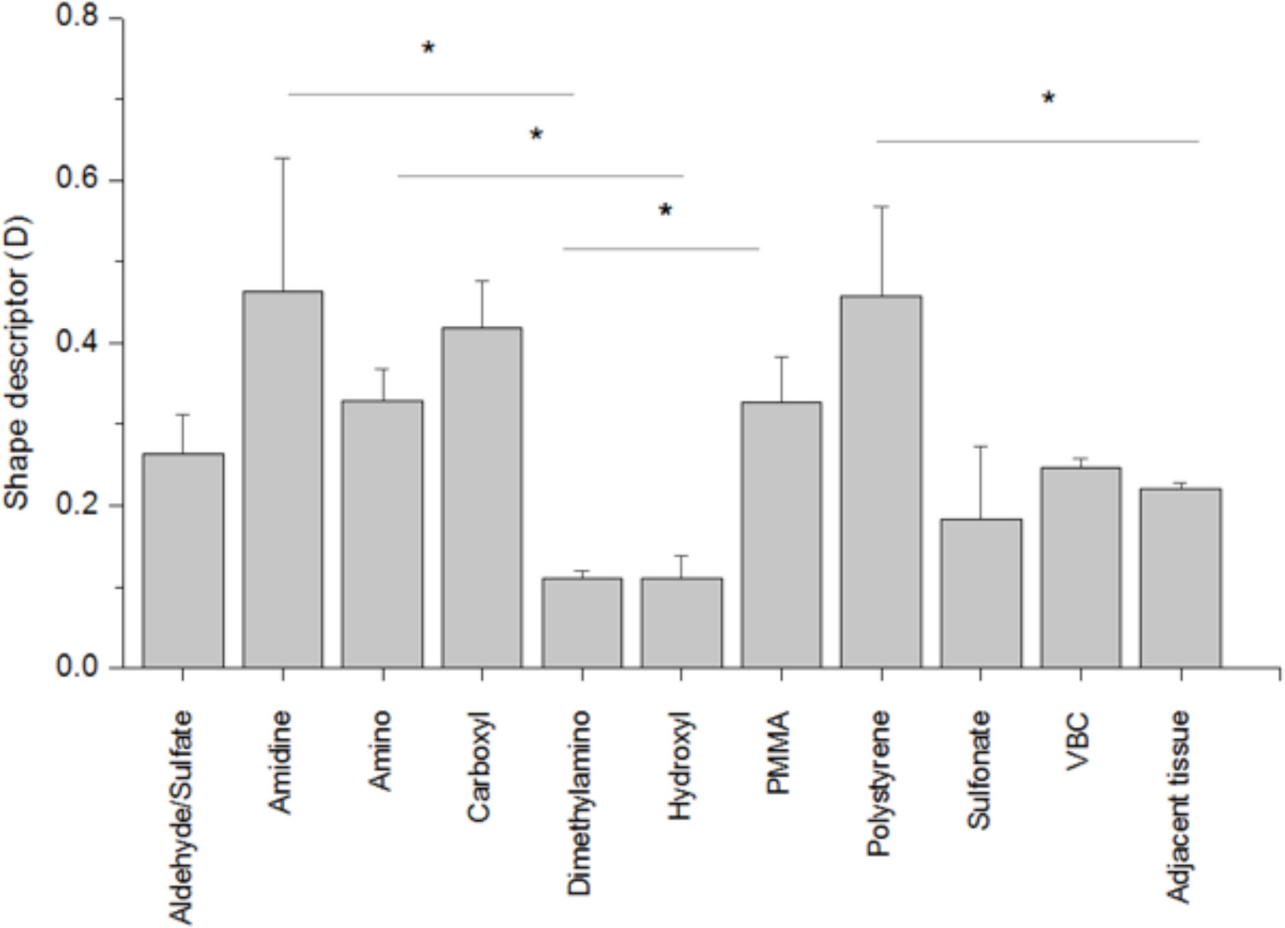
Shape descriptors of fibrotic capsules surrounding subcutaneously implanted polystyrene particles. For *D* = 1 collagen orientation is uniaxial, while for collagen fibers with *D* = 0 orientation is completely random. All experiments were done in quadruplicate. Results are expressed as the mean value ± standard deviation. (*) indicates p<0.05.

To quantify the shapes of polarimetry profiles, *PA*, *D*, and *e* values were calculated (S1 Fig, for *PA* and *e* and Fig 4 for *D*). *D* = 1 represents a uniaxial collagen orientation, while orientation is completely random for collagen fibers with *D* = 0. Amino, carboxyl, and polystyrene had the highest values for all three descriptors. *D* and *e* values for tissue sections with amidine particles (0.464 ± 0.020 and 0.482 ± 0.101) were high compared to the control tissue sections. The *D* descriptor for tissue sections with amidine, carboxyl, and polystyrene particles were significantly higher compared to the control tissue sections, which was a section of skin not injected with particles (p < 0.05) (Fig 4). Aldehyde/sulfate, sulfonate, dimethylamino, hydroxyl, VBC and PMMA particles have collagen orientation comparable to the control.

**Fig 5:**
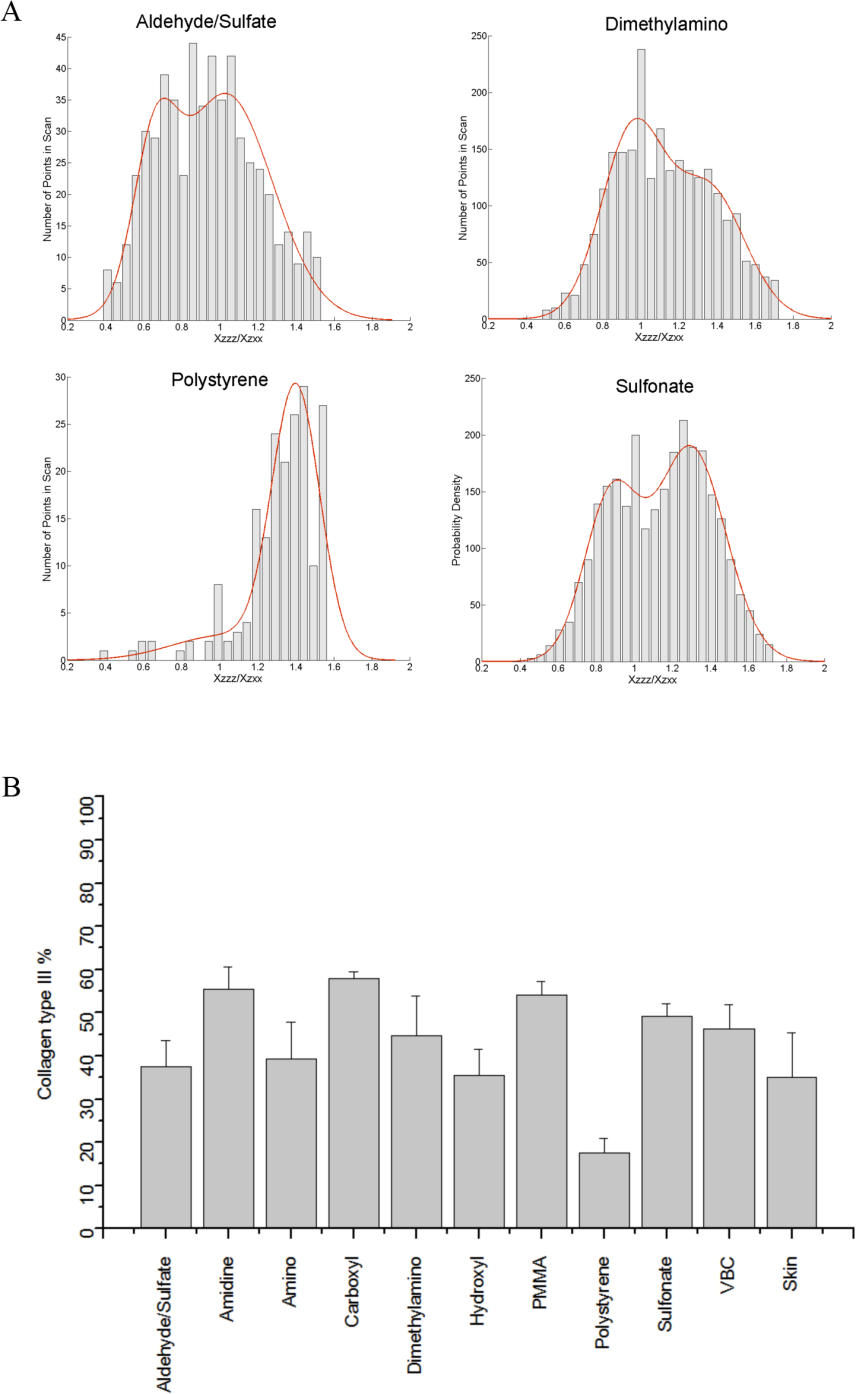
A) Histograms of $\frac{\chi_{\text{zzz}}}{\chi_{\text{zxx}}}$ values obtained for tissue sections of subcutaneously implanted aldehyde/sulfate, amidine, amino and dimethylamino polystyrene particles. The bars represent experimentally acquired data. The red line is the bimodal Gaussian fit to the data. B) Collagen type III content of fibrotic capsules surrounding subcutaneously implanted polystyrene particles.

**Fig 6:**
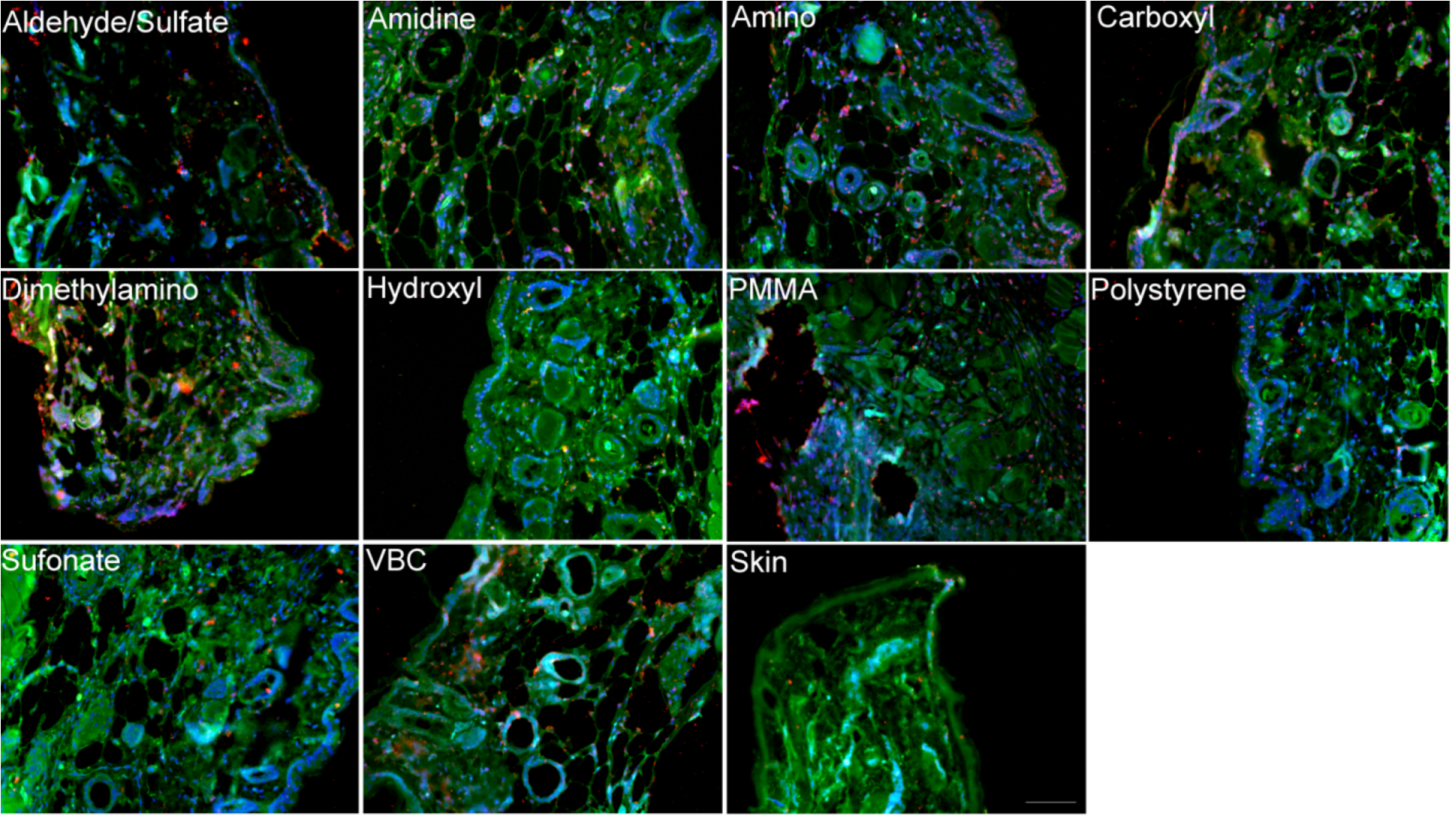
Representative immunofluorescence images of mouse tissues subcutaneously injected with various polystyrene particles. Green is collagen type I, red is collagen type III, and blue is DAPI. The scale bar represents 100 μm.

Structural inhomogenities were also quantified using SHG imaging. The gradient line obtained in Fig 1B was used to estimate the collagen type III % concentration in the fibrotic capsule surrounding the modified polystyrene particles (Fig 5A, S2 Table). Jain and co-workers [38] have previously examined matrigel, laminin, and collagen IV and found no SHG signal. This was further demonstrated by Nguyen-Ngoc and Ewald [39], who also reported an absence of signal from Matrigel. The analysis in this study assumes that the SHG signal arises from collagen type I and type III. Collagen type III peaks were observed for all tissue sections injected with polystyrene particles. The collagen type I and type III histogram peak assignments are given in S2 Table. Fibrotic capsules surrounding the unmodified polystyrene particles had the lowest content of collagen type III (17.55 ± 3.45%). Control tissue sections showed 35.05 ± 10.30% of collagen type III, which agrees with previously obtained results [11]. Amidine, carboxyl, and PMMA particles showed the highest level of collagen type III in their fibrotic capsule with around 50% of collagen type III (Fig 5B). The fibrotic capsules surrounding the amino, hydroxyl, and aldehyde/sulfate implanted particles had similar collagen type III content as the control tissue. Tissue sections were also stained for the presence of collagen types I and III (Fig 6) for qualitative confirmation of the SHG results. In the fibrous capsules surrounding dimethylamino and carboxyl, higher collagen type III content was noticed compared to polystyrene, hydroxyl, and the control skin tissue. While aldehyde/sulfate, amidine, amino, PMMA, and VBC showed similar level of collagen type III. Due to differences in quantum yields and molar absorptivities between the different fluorophores used, only qualitative descriptions of the immunohistochemistry images were possible.

## Discussion

Collagen orientation plays a crucial role in the growth and repair of the tissue that can dictate a successful outcome of an implanted device. Thin, well oriented collagen fibers in most cases are associated with scarring, which can lead to failure of the implanted device due to low vascular density [18,40]. It has been shown that the amount of collagen deposited and its organization depends on the material *in vitro* and *in vivo* [34,41]. Collagen fiber orientation analysis exhibited different patterns for all polystyrene beads, demonstrating the ability of surface chemistry to alter *in vivo* fate.

Here, we used SHG radar graphs for analyzing collagen fiber orientation in the fibrotic capsule surrounding implanted polystyrene particles. Quantifying the shape of the polarimetry profiles further describes the alignment of the collagen. Yasui *et al*. have quantified collagen orientation by calculating the PA for different collagenous structures [30,40]. This anisotropy parameter has been found to be useful in characterizing collagen orientation [26,42]. We found that for our work, the PA value cannot precisely describe asymmetrically shaped polarimetry profiles and distinguish between differently shaped profiles. For example, polarimetry profiles for carboxyl and dimethylamino in Fig 4B have very similar PA values (0.029 ± 0.002 and 0.022 ± 0.003, respectively); however, the profiles for both particles are quite different, as seen by visual inspection of the polarimetry profiles and through examining the D values of 0.38 ± 0.01 and 0.15 ± 0.01, respectively. The same trend can be observed for sulfonate and VBC particles. The eccentricity of the polarimetry profile shows a slight correlation with shape factor D (*R* ^2^ = 0.720). We chose to use the D values to interpret our images since this parameter measures how close the polarimetry profile is to a perfect circle meaning that the collagen is isotropic, whereas the eccentricity parameter examines whether a shape is circular or elliptical.

To determine the composition of collagen deposited in fibrotic capsules, collagen type III content was quantified. Collagen type I is known to confer tensile strength in connective tissues, while collagen type III has a thinner diameter and is mostly found during the early stages of wound healing. Collagen type III is also responsible for fibrillogensis [13,43,44]. Here, we showed the ability of SHG microscopy to assess the composition of a fibrous capsule using the nonlinear susceptibility tensor ratio $\frac{\chi_{zzz}}{\chi_{zxx}}$. Information about collagen type III concentration in the fibrous capsule surrounding the various polystyrene beads was extracted from the gradient curve obtained from collagen gels with defined collagen combinations. Our estimated values of % collagen III showed a wide range across the samples. There are some particles having slightly over 50% of collagen type III and particles with the same concentration as the control tissue. There are several factors that can affect collagen type III concentration. Increased collagen type III secretion is usually observed in acutely or chronically inflamed tissues [45]. It has also been shown that co-culturing fibroblast cells with macrophages increases collagen type III secretion [46]. In tissue/biomaterial interactions, it is thought that the deposition of different collagen types depends on blood elements such as platelet-derived growth factor interactions with the surface of the material and the interaction of inflammatory cells [47].

The higher ratio of collagen type III over collagen type I that we observed for some particles might be associated with prolonged inflammation and thicker capsule walls [20,43]. In general, there was no correlation between collagen type III content and collagen orientation in fibrous capsule (R^2^ = 0.000), which may be due to different cytokine or growth factor expression profiles induced by these particles [35,47,48]. There was a correlation between the fibrotic capsule thickness and collagen type III content (R^2^ = 0.762), which is expected based on previous results showing that a decrease in collagen type III leads to excessive scar formation [15]. Collagen orientation had no correlation with fibrotic capsule thickness (R^2^ = 0.001).

**Fig 7:**
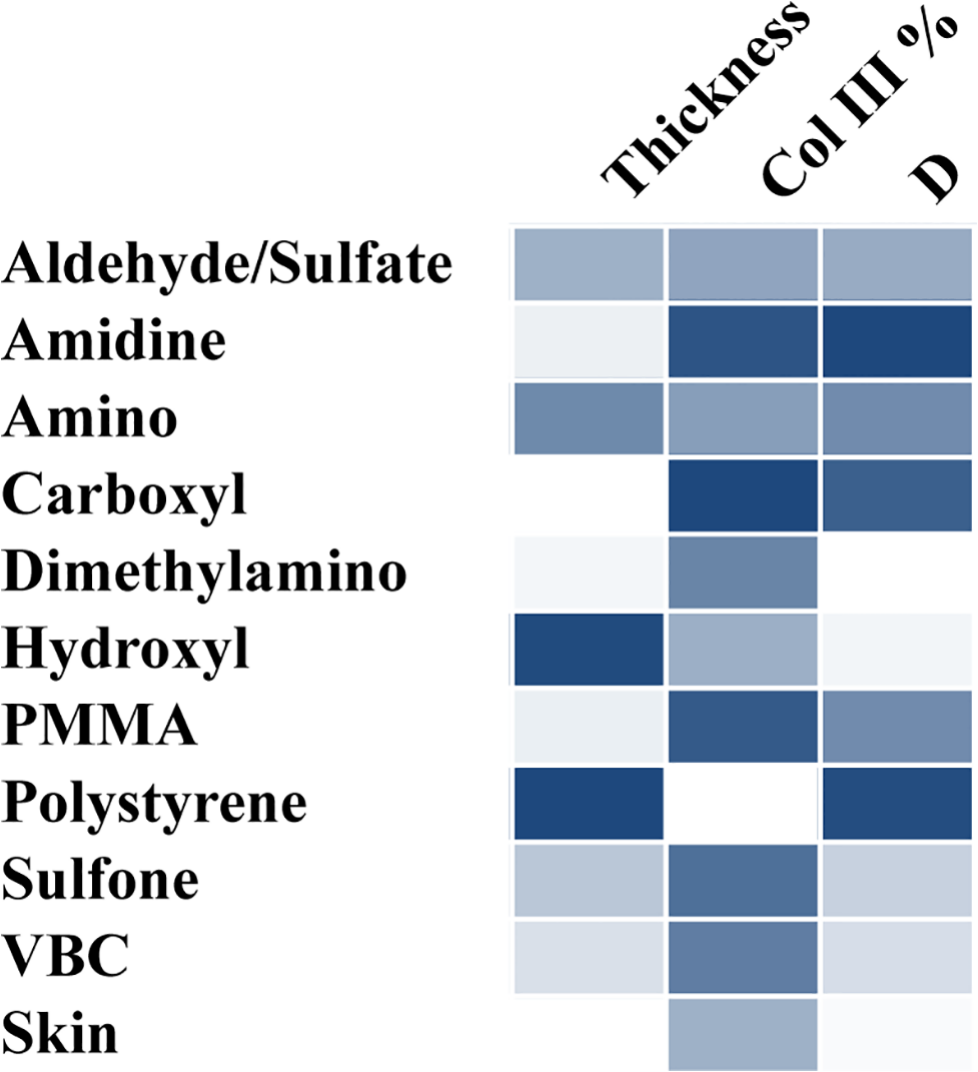
Heat map comparison of fibrotic capsule thickness, % of collagen type III, and the D shape factor.

One of the goals for many tissue engineering and biomaterial applications is to induce healthy tissue formation [49]. As discussed above, thin, isotropic collagen surrounding the implant with similar collagen type III content as healthy skin is the ideal scenario for subcutaneous implants. A comparison of the effect of polymer surface chemistry studied here can be seen in Fig 7 as a heat map. For the control skin sample, the fibrotic capsule thickness was omitted. Dimethylamino functionalized polystyrene appears to induce a thin layer of isotropic collagen. Based on Fig 7, the collagen synthesized around the dimethylamino implant appears to be higher than the healthy skin sample. However, in examining the values and their associated errors in Fig 5B, dimethylamino is statistically similar to the healthy skin, suggesting that these particles can be effective in successfully resolving the foreign body response and can stimulate organization of collagen similar to normal skin. On the opposite end of the spectrum, amino and polystyrene particles elicit a thick fibrotic capsule composed of anisotropic collagen. All of the particles yielded a continuum of responses, demonstrating the ability to engineer the foreign body response to an implanted material.

## Conclusions

The results of this study demonstrate the ability of SHG microscopy to estimate the fibrotic capsule structural inhomogenities and collagen orientation. This is important progress in the ability to modulate the foreign body response through biomaterials. We calculated the anisotropy of the collagen orientation using traditional polarization anisotropy and two shape factors. Since unwounded collagen in skin has an isotropic distribution, polarimetry profiles were quantified using a shape descriptor that measures how similar the radar plot is to a perfect circle. A wide range of collagen type III composition, collagen fiber orientation, and fibrotic capsule thickness was observed for all ten of the materials studied here. Dimethylamino functionalized particles induced a thin fibrotic capsule that was randomly oriented with a collagen type III content similar to healthy skin. The unmodified polystyrene resulted in a thick fibrotic capsule that was well-aligned and had a very low collagen type III content, all of which are undesirable outcomes. These findings and using SHG microscopy to analyze both the collagen orientation and composition of the fibrotic capsule have the potential to improve the performance of implanted medical devices where fibrotic capsule formation is undesirable.

## Supporting Information

S1 Fig Eccentricity and polarization anisotropy values for tissue sections with different PS particles. (TIF)

S1 Table Peak position values from bimodal Gaussian distribution of different gel mixtures. (TIF)

S2 TablePeak position values of $\frac{\chi_{zzz}}{\chi_{zxx}}$ from bimodal Gaussian distribution for tissue sections with different PS particles.(TIF)

## References

[1] Anderson JM, Rodriguez A, Chang DT. Foreign body reaction to biomaterials. Semin Immunol. 2008;20: 86–100. 10.1016/j.smim.2007.11.004 18162407 PMC2327202

[2] Anderson JJM. Biological responses to materials. Annu Rev Mater Res. 2001;31: 81–110. Available: http://www.annualreviews.org/doi/pdf/10.1146/annurev.matsci.31.1.81

[3] Luttikhuizen DT, Harmsen MC, Van Luyn MJA. Cellular and molecular dynamics in the foreign body reaction. Tissue Eng. 2006;12: 1955–1970. 10.1089/ten.2006.12.1955 16889525

[4] Tang L, Jennings TA, Eaton JW. Mast cells mediate acute inflammatory responses to implanted biomaterials. Proc Natl Acad Sci U S A. 1998;95: 8841–8846. 10.1073/pnas.95.15.8841 9671766 PMC21164

[5] Jenney CR, Anderson JM. Adsorbed serum proteins responsible for surface dependent human macrophage behavior. J Biomed Mater Res. 2000;49: 435–47. Available: http://www.ncbi.nlm.nih.gov/pubmed/10602077 10602077 10.1002/(sici)1097-4636(20000315)49:4<435::aid-jbm2>3.0.co;2-y

[6] Sperling C, Fischer M, Maitz MF, Werner C. Blood coagulation on biomaterials requires the combination of distinct activation processes. Biomaterials. Elsevier Ltd; 2009;30: 4447–56. 10.1016/j.biomaterials.2009.05.044 19535136

[7] Broughton G, Janis JE, Attinger CE. The basic science of wound healing. Plast Reconstr Surg. 2006;117: 12S–34S. 10.1097/01.prs.0000225430.42531.c2 16799372

[8] Hinz B. Formation and function of the myofibroblast during tissue repair. J Invest Dermatol. 2007;127: 526–37. 10.1038/sj.jid.5700613 17299435

[9] Bygd HC, Forsmark KD, Bratlie KM. The significance of macrophage phenotype in cancer and biomaterials. Clin Transl Med. 2014;3. 10.1186/s40169-014-0041-2 PMC4884036 26932379

[10] Vos P De, Faas MM, Strand B, Calafiore R, de Vos P. Alginate-based microcapsules for immunoisolation of pancreatic islets. Biomaterials. 2006;27: 5603–5617. 10.1016/j.biomaterials.2006.07.010 16879864

[11] Cheung DT, Benya PD, Perelman N, DiCesare PE NM. A highly specific and quantitative method for determining type III/I collagen ratios in tissues. Matrix. 1990;10: 164–71. 2215356 10.1016/s0934-8832(11)80165-4

[12] Gay S, Vijanto J, Raekallio J, Penttinen R. Collagen types in early phases of wound healing in children. Acta Chir Scand. 1978;144: 205–211. 360747

[13] Stuart K, Panitch A. Characterization of gels composed of blends of collagen I, collagen III, and chondroitin sulfate. Biomacromolecules. 2009;10: 25–31. 10.1021/bm800888u 19053290

[14] Liu SH, Yang RS, al-Shaikh R, Lane JM. Collagen in tendon, ligament, and bone healing. A current review. Clin Orthop Relat Res. 1995; 265–278. 7671527

[15] Volk SW, Wang Y, Mauldin E a, Liechty KW, Adams SL. Diminished type III collagen promotes myofibroblast differentiation and increases scar deposition in cutaneous wound healing. Cells Tissues Organs. 2011;194: 25–37. 10.1159/000322399 21252470 PMC3128157

[16] Verhaegen PDHM, Marle J Van, Kuehne A, Schouten HJ, Gaffney E a, Maini PK, et al Collagen bundle morphometry in skin and scar tissue: a novel distance mapping method provides superior measurements compared to Fourier analysis. J Microsc. 2012;245: 82–9. 10.1111/j.1365-2818.2011.03547.x 21919907

[17] Martin P. Wound Healing—Aiming for Perfect Skin Regeneration. Science (80-). 1997;276: 75–81. 10.1126/science.276.5309.75 9082989

[18] Van Zuijlen PPM, Ruurda JJB, van Veen HA, van Marle J, van Trier AJM, Groenevelt F, et al Collagen morphology in human skin and scar tissue: no adaptations in response to mechanical loading at joints. Burns. 2003;29: 423–31. Available: http://www.ncbi.nlm.nih.gov/pubmed/12880721 12880721 10.1016/s0305-4179(03)00052-4

[19] Chavrier C, Couble ML, Hartmann DJ. Qualitative study of collagenous and noncollagenous glycoproteins of the human healthy keratinized mucosa surrounding implants. Clin Oral Implants Res. 1994;5: 117–124. 7827225 10.1034/j.1600-0501.1994.050301.x

[20] Jacob JT, Gebhardt BM, Lewando J. Synthetic scleral reinforcement materials. II. Collagen types in the fibrous capsule. J Biomed Mater Res. 1996;32: 181–186. 10.1002/(SICI)1097-4636(199610)32:2<181::AID-JBM5>3.0.CO;2-P 8884493

[21] Shannon C, Thull R, Von Recum A. Types I and III collagen in the tissue capsules of titanium and stainless-steel implants. J Biomed Mater Res. 1997;34: 401–408. 10.1002/(SICI)1097-4636(19970305)34:3<401::AID-JBM15>3.0.CO;2-I 9086410

[22] Chen X, Nadiarynkh O, Plotnikov S, Campagnola PJ. Second harmonic generation microscopy for quantitative analysis of collagen fibrillar structure. Nat Protoc. Nature Publishing Group; 2012;7: 654–69. 10.1038/nprot.2012.009 PMC4337962 22402635

[23] Campagnola PJ, Loew LM. Second-harmonic imaging microscopy for visualizing biomolecular arrays in cells, tissues and organisms. Nat Biotechnol. 2003;21: 1356–60. 10.1038/nbt894 14595363

[24] Francesco S. Pavone; Paul J. Campagnola, editor. Socond Harmonic Generation Imaging. Taylor & Francis; 2013. doi: K12222

[25] Tiaho F, Recher G, Rouède D. Estimation of helical angles of myosin and collagen by second harmonic generation imaging microscopy. Opt Express. 2007;15: 12286–12295. 10.1364/OE.15.012286 19547597

[26] Hompland T, Erikson A, Lindgren M, Lindmo T, de Lange Davies C. Second-harmonic generation in collagen as a potential cancer diagnostic parameter. J Biomed Opt. 2014;13: 054050 10.1117/1.2983664 19021430

[27] Ambekar R, Lau T-Y, Walsh M, Bhargava R, Toussaint KC. Quantifying collagen structure in breast biopsies using second-harmonic generation imaging. Biomedical Optics Express. 2012. p. 2021. 10.1364/BOE.3.002021 PMC3447546 23024898

[28] Su P-J, Chen W-L, Chen Y-F, Dong C-Y. Determination of collagen nanostructure from second-order susceptibility tensor analysis. Biophys J. Biophysical Society; 2011;100: 2053–62. 10.1016/j.bpj.2011.02.015 PMC3077698 21504742

[29] Gavin HP. The Levenberg-Marquardt method for nonlinear least squares curve-fitting problems Department of Civil and Environmental Engineering, Duke University. 2013.

[30] Yasui T, Tohno Y, Araki T. Determination of collagen fiber orientation in human tissue by use of polarization measurement of molecular second-harmonic-generation light. Appl Opt. 2004;43: 2861–7. 15143809 10.1364/ao.43.002861

[31] Yuan Z, Li F, Zhang P, Chen B. Description of shape characteristics through Fourier and wavelet analysis. Chinese J Aeronaut. Chinese Society of Aeronautics and Astronautics; 2014;27: 160–168. 10.1016/j.cja.2013.07.011

[32] Tuer AE, Akens MK, Krouglov S, Sandkuijl D, Wilson BC, Whyne CM, et al. Hierarchical model of fibrillar collagen distribution for polarization-resolved SHG microscopy. Periasamy A, König K, So PTC, editors. Proc SPIE. 2013;8588: 85881O. 10.1117/12.2000724

[33] Chen W-L, Li T, Su P, Chou C-K, Fwu PT, Lin S-J, et al Second harmonic generation X tensor microscopy for tissue imaging. Appl Phys Lett. 2009;94: 183902 10.1063/1.3132062

[34] Curtis ASG, Forrester J V., McInnes C, Lawrie F. Adhesion of cells to polystyrene surfaces. J Cell Biol. 1983;97: 1500–1506. 10.1083/jcb.97.5.1500 6355120 PMC2112677

[35] Zhang L, Cao Z, Bai T, Carr L, Ella-Menye J-R, Irvin C, et al Zwitterionic hydrogels implanted in mice resist the foreign-body reaction. Nat Biotechnol. Nature Publishing Group; 2013;31: 553–6. 10.1038/nbt.2580 23666011

[36] VandeVord PJ, Matthew HWT, DeSilva SP, Mayton L, Wu B, Wooley PH. Evaluation of the biocompatibility of a chitosan scaffold in mice. J Biomed Mater Res. 2002;59: 585–90. Available: http://www.ncbi.nlm.nih.gov/pubmed/11774317 11774317 10.1002/jbm.1270

[37] Park S, Park M, Kim BH, Lee JE, Park HJ, Lee SH, et al Acute suppression of TGF-ß with local, sustained release of tranilast against the formation of fibrous capsules around silicone implants. J Control Release. Elsevier B.V.; 2015;200: 125–137. 10.1016/j.jconrel.2014.12.021 25528612

[38] Brown E, Mckee T, diTomaso E, Pluen A, Seed B, Boucher Y, et al Dynamic imaging of collagen and its modulation in tumors in vivo using second-harmonic generation. Nat Med. 2003;9: 796–800. 12754503 10.1038/nm879

[39] Nguyen-Ngoc K V., Ewald a. J. Mammary ductal elongation and myoepithelial migration are regulated by the composition of the extracellular matrix. J Microsc. 2013;251: 212–223. 10.1111/jmi.12017 23432616 PMC3978143

[40] Yasui T, Tohno Y, Araki T. Characterization of collagen orientation in human dermis by two-dimensional second-harmonic-generation polarimetry. J Biomed Opt. 2004;9: 259–64. 10.1117/1.1644116 15065889

[41] Marinucci L, Lilli C, Guerra M, Belcastro S, Becchetti E, Stabellini G, et al Biocompatibility of collagen membranes crosslinked with glutaraldehyde or diphenylphosphoryl azide: An in vitro study. J Biomed Mater part A. 2003;67: 504–509. 10.1002/jbm.a.10082 14566791

[42] Nadiarnykh O, LaComb RB, Brewer M a, Campagnola PJ. Alterations of the extracellular matrix in ovarian cancer studied by Second Harmonic Generation imaging microscopy. BMC Cancer. 2010;10: 94 10.1186/1471-2407-10-94 20222963 PMC2841668

[43] Stadelmann WK, Digenis AG, Tobin GR. Physiology and healing dynamics of chronic cutaneous wounds. American Journal of Surgery. 1998 10.1016/S0002-9610(98)00183-4 9777970

[44] Dale PD, Sherratt JA, Maini PK. A mathematical model for collagen fibre formation during foetal and adult dermal wound healing. Proc Biol Sci. 1996;263: 653–660. 10.1098/rspb.1996.0098 8677263

[45] Bailey AJ, Sims TJ, Le Lous M, Bazin S. Collagen polymorphism in experimental granulation tissue. Biochem Biophys Res Commun. 1975;66: 1160–1165. 10.1016/0006-291X(75)90480-5 1191284

[46] Ploeger DT, Hosper N a, Schipper M, Koerts J a, de Rond S, Bank R a. Cell plasticity in wound healing: paracrine factors of M1/ M2 polarized macrophages influence the phenotypical state of dermal fibroblasts. Cell Commun Signal. 2013;11: 29 10.1186/1478-811X-11-29 23601247 PMC3698164

[47] Kambic HE, Kantrowitz A SP. Vascular Graft Update. 1986.

[48] Akilbekova D, Philiph R, Graham A, Bratlie KM. Macrophage reprogramming: influence of latex beads with various functional groups on macrophage phenotype and phagocytic uptake in vitro. J Biomed Mater Res Part A. 2015; 10.1002/jbm.a.35169 24639060

[49] Badylak SF, Weiss DJ, Caplan A, MacChiarini P. Engineered whole organs and complex tissues. Lancet. Elsevier Ltd; 2012;379: 943–952. 10.1016/S0140-6736(12)60073-7 22405797

