# Supplementary figures and images for: Quantitative Characterization of Collagen in the Fibrotic Capsule Surrounding Implanted Polymeric Microparticles through Second Harmonic Generation Imaging
Source: PLoS One. 2015 Jun 30;10(6):e0130386. doi: 10.1371/journal.pone.0130386 (PMC4488378; doi:10.1371/journal.pone.0130386)

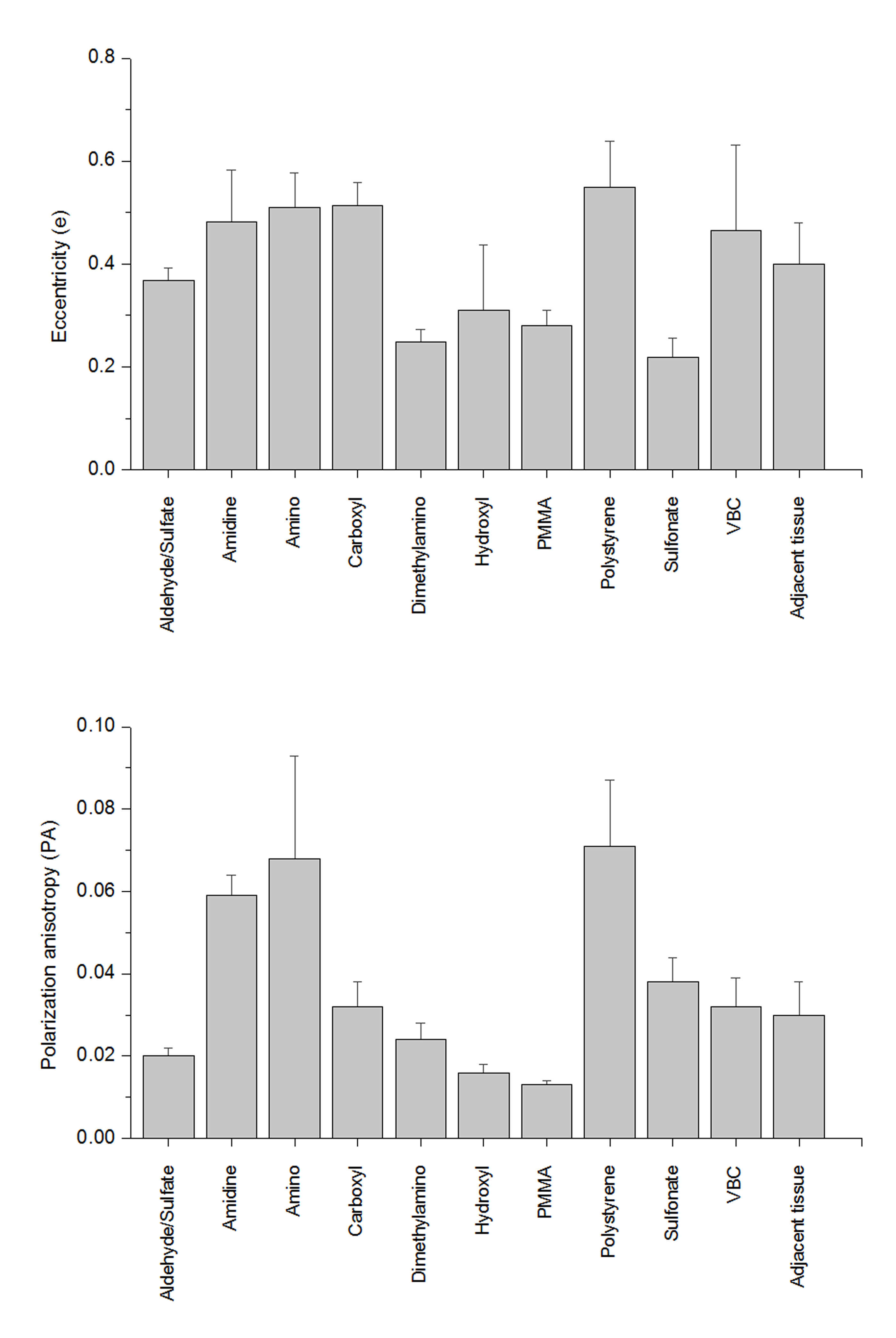

Supplement: S1 Fig — (TIF) [file pone.0130386.s001.tif]

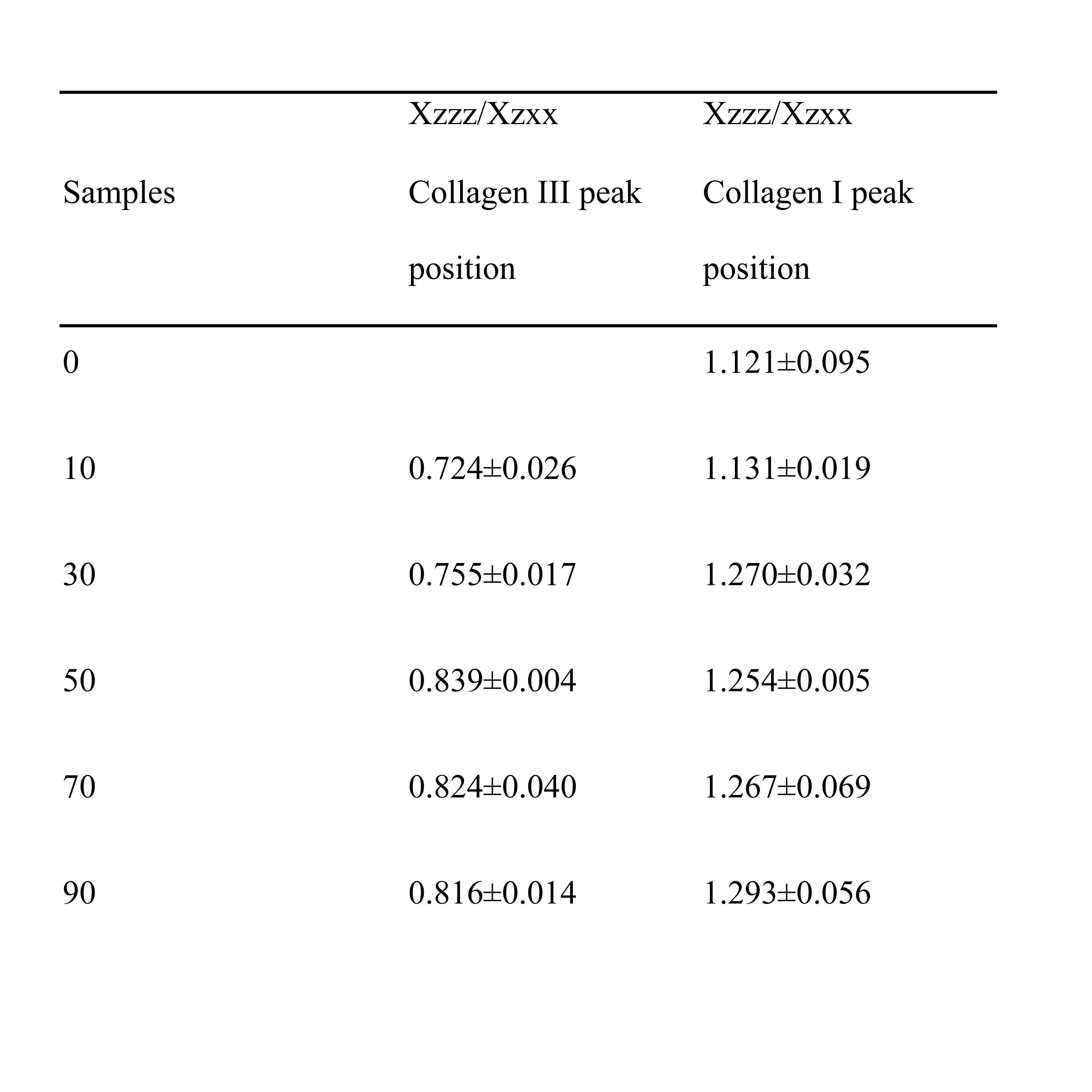

Supplement: S1 Table — (TIF) [file pone.0130386.s002.tif]

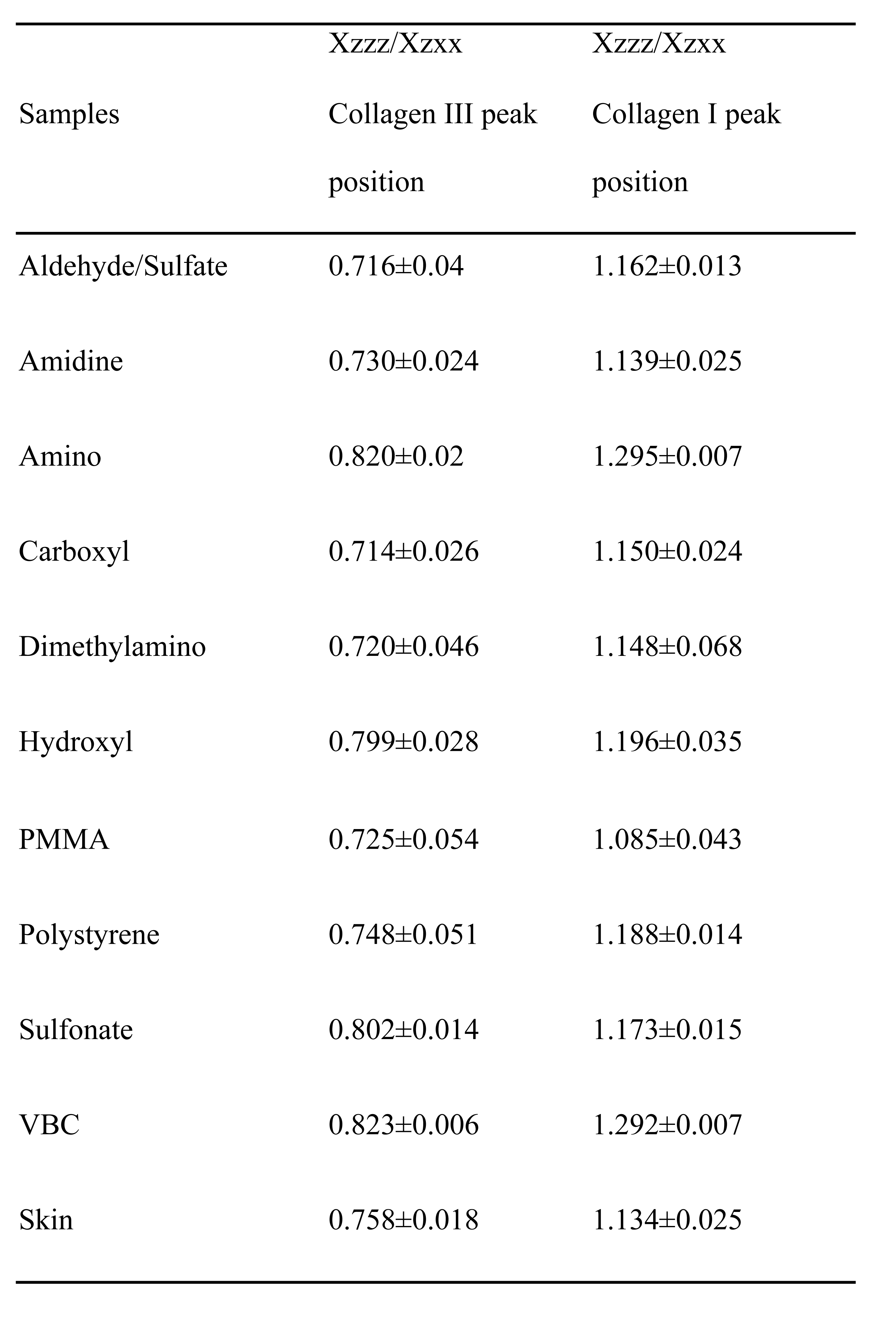

Supplement: S2 Table — (TIF) [file pone.0130386.s003.tif]
